# A cohort study of men infected with COVID-19 for presence of SARS-CoV-2 virus in their semen

**DOI:** 10.1007/s10815-021-02119-y

**Published:** 2021-03-03

**Authors:** Corey A. Burke, AB Skytte, Saghar Kasiri, Dixie Howell, Zamip P. Patel, Mark P. Trolice, Sijo J. Parekattil, Scott F. Michael, Lauren M. Paul

**Affiliations:** 1 Cryos International USA, 2200 N Alafaya TR, Suite 550, Orlando, FL 32826 USA; 2 Cryos International DK, Vesterbro Torv 3, 5th Floor, Aarhus C, DK-8000 UK; 3 Advent Health Urology, Orlando, FL USA; 4 Fertility Care: The IVF Center Orlando, 5901 Brick Cr, Winter Park, FL 32792 USA; 5 Avant Concierge Urology, 15548 W. Colonial Dr, Winter Garden, FL 34787 USA; 6 Department of Biological Sciences, Florida Gulf Coast University, 10501 FGCU Blvd South, Ft. Myers, FL 33965 USA

**Keywords:** Semen, Sperm, COVID-19, SARS-CoV-2

## Abstract

**Introduction:**

Whether severe acute respiratory syndrome coronavirus 2 (SARS-CoV-2) can be detected in semen and transmitted sexually is a vital question that has, thus far, been inconclusive. Prior studies, with limited numbers, have included men in various stages of infection with most in the recovery phase of the illness. The timing of test results and severity of illness has made recruiting study participants a significant challenge. Our pilot study will examine semen from men with a recent diagnosis of COVID-19 as well as those in the convalescent phase to determine if SARS-CoV-2 can be detected and its relationship, if any, with the severity of the disease.

**Methods:**

Eighteen men with a median age of 32 (range, 24-57) who tested positive for COVID-19 by rt-PCR analysis were enrolled and provided a semen sample. The study group demonstrated symptoms of COVID-19 ranging from asymptomatic to moderate and none required hospitalization. Samples were subjected to viral RNA extraction and then processed by real-time RT-PCR using the US Centers for Disease Control and Prevention (CDC, USA) panel of 2019-Novel Coronavirus (2019-nCoV) primers and probes to detect the presence of SARS-CoV-2 RNA.

**Results:**

Length of time from diagnosis to providing a specimen ranged from 1 to 28 days (median, 6 days). Fifteen participants were symptomatic and three were asymptomatic, including recovering men, at the time of semen collection. No SARS-CoV-2 was detected in any of the semen samples.

**Conclusion:**

Based on these preliminary results and consistent with prior findings, we suggest SARS-CoV-2 is not present in semen during the acute or convalescent phase of COVID-19.

## Introduction

The pandemic of SARS-CoV-2 has raised many questions regarding the impact of the virus on organ systems in the human body. Analogous to the concerns during the Zika outbreak, recent findings reveal that angiotensin converting enzyme 2 (ACE 2), which is found in testicular tissue, plays a major role in the pathogenesis of SARS-CoV-2 [1].

The possibility of sexual transmission of the virus is of major interest, specifically to those in the field of reproductive medicine. In March 2020, both American Society for Reproductive Medicine and European Society of Human Reproduction and Embryology provided guidance to all IVF centers to discontinue offering ART services to conserve resources of PPE as well as reduce the risk of SARS-CoV-2 transmission [2, 3].

Previous studies have focused on men who were in various stages of recovery from infection and have shown no evidence of SARS-CoV-2 in semen. One of these studies by Holtmann et al. looked at 18 patients who had recovered from SARS-CoV-2 infection and found no evidence of viral RNA in their semen [4]. Additionally, the authors studied ACE2 and TMPRSS2 within the testicles and determined viral entry into host cells in the human testicle is unlikely. Another study by Pan et al. examined semen samples from 34 men who had recovered from SARS-CoV-2 (median 31 days post infection) and demonstrated no viral RNA in semen [5].

A definitive determination for the presence of SARS-CoV-2 in semen would have major implications for assisted reproductive therapies (ART) and donor sperm banks. Previous studies have examined semen in men recovering from SARS-CoV-2 rather than during the acute phase of the disease primarily because of the delay in receiving test results. Many infected with SARS-CoV-2 wait up to 2 weeks for results due to testing laboratories being overwhelmed with samples thereby converting to asymptomatic in the convalescent phase, though recent advances in testing techniques have shortened gaps between testing and diagnosis.

Our pilot study will examine semen from men with a recent diagnosis of COVID-19 as well as those in the convalescent phase to determine if SARS-CoV-2 can be detected and its relationship, if any, with the severity of the disease.

## Materials and methods

The samples were collected at the laboratory of Cryos International, Orlando, FL, between March 10, 2020, and October 29, 2020, and approved by the Western Institutional Review Board (20200888). Eligibility criteria were men between the ages of 18 and 60, with an intact reproductive tract, and who tested positive for SARS-CoV-2 RNA 2 by nasopharyngeal swabs using rt-PCR, regardless of symptomatology.

Exclusion criteria included men who had a vasectomy, men with a urologic condition that inhibits ejaculation, taking testosterone or hormonal treatment that can impact semen production, undergoing chemotherapy, on immunosuppressant medication, and with a concurrent acute or chronic infection.

Study participants were instructed to provide a semen specimen free of contamination by cleaning the penis and hands as well as avoiding saliva during collection. Specimens were collected in a sterile collection cup and allowed to liquefy before aliquoting into cryovials. The specimens were frozen using a controlled rate freezer and stored under liquid nitrogen at −196 °C. Specimens were transported to Florida Gulf Coast University, Fort Myers, FL, using a dry shipper to maintain −196 °C.

Upon arrival, samples were stored at −80 °C until processed. When processed, samples were thawed and 200 μL aliquoted from the original cryovial. Aliquot was centrifuged at 3500 rpm for 5 min in a table-top centrifuge (Grant PCV-2400 Combined Centrifuge/Vortex Mixer, Grant Instruments, Cambridge, UK) to spin-out any cellular material or debris. Then 140 μL supernatant from centrifuged samples was transferred into a new tube. Any remaining, unused sample was frozen back and stored at −80 °C. Viral RNA was extracted from the 140 μL collected supernatant following the protocol outlined in the QIAamp Viral RNA Mini Kit (Qiagen, Hilden, Germany). A water-only control (RNA Blank) was included with each round of extraction to check for RNA contamination. Sample viral RNA extractions were eluted in 60 μL nuclease-free water and stored at 4 °C (24 h maximum) until ready for qRT-PCR application.

Reactions for real-time RT-PCR were assembled using GoTaq® Probe 1-Step RT-qPCR System (Promega, Madison, WI). Twenty-microliter reactions were prepared, using 5 μL of RNA extract template per reaction. Each sample extracted, including controls, was tested in triplicate. Additional water-only, no template control (NTC), was added to check for contamination of master-mix and/or individual samples during loading. The US Centers for Disease Control and Prevention (CDC, USA) panel of 2019-Novel Coronavirus (2019-nCoV) primers and probes, specifically for SARS-CoV-2 targets N1 and N2, were utilized in recommended concentrations from IDT-DNA, 2019-nCoV Research-Use-Only Kit (Integrated DNA Technologies, Coralville, IA). *A link to CDC panel of primers and probes sequences is listed at the end of “Materials and methods” section. A dilution series of Sars-CoV-2_USA_WA1 N-gene transcript RNA (shared by Dr. Nathan Grubaugh at Yale University, New Haven, CT) served as a positive control and to create duplicate standards for assessing genome copy number.

Reactions were run on CFX96 Touch Real-Time PCR Detection System (Bio-Rad Laboratories, Hercules, CA) under the following conditions: Reverse transcription at 45 °C for 15 min followed by single cycle of 95 °C for 2 min, then 45 cycles of PCR with denaturation at 95 °C for 10 s and combined annealing/extension at 55 °C for 45 s.

Limit of detection was determined to be 20 genome copies of positive transcript RNA. Control RNA stock was serially diluted tenfold and then verified by qRT-PCR with both N1 and N2 primer sets.

Samples’ amplification curves and Cp values were compared to those of positive control standards in order to determine whether positive or negative for presence of SARS-CoV-2 RNA.

To assess the potential for qRT-PCR inhibitors from the samples, positive control reactions with N-gene transcript RNA were spiked with sample RNA extracts.

*Link to CDC panel of 2019-Novel Coronavirus (2019-nCoV) primers and probes:

https://www.cdc.gov/coronavirus/2019-ncov/downloads/rt-pcr-panel-primer-probes.pdf

## Results

Eighteen men between the ages of 24 and 57, with a median age of 32 years, were enrolled as study participants. The time from virus detection to semen collection ranged from 1 to 28 days (median 6 days). Eleven men were symptomatic for COVID-19 at the time of semen collection. One man remained asymptomatic and tested positive 6 days prior to and 5 days after collection. Two men, who were symptomatic, tested positive at 14 and 16 days prior to providing a semen sample but were not retested. Three men were diagnosed 21 to 28 days prior to semen collection when they were recovered from symptoms. One man was diagnosed 28 days prior to collection and remained symptomatic.

The severity of disease was ranked as asymptomatic, mild, moderate, or severe based on self-reporting with severe designating the requirement of hospitalization. One man was classified as asymptomatic, two were mild, fifteen were moderate, and zero was severe.

Fever was the most common symptom (89%) with the distribution of all symptoms shown in Table 1, followed by body aches (56%), and loss of smell (56%). Headache (33%), fatigue (33%), loss of taste (33%), and respiratory issues (33%) were second. Other symptoms included eye pain (11%), and flu-like symptoms. Irrespective of symptoms at the time of semen collection, no semen sample tested positive for SARS-CoV-2.

**Table 1:** Enrolled patients’ demographics (age), time of diagnosis by nasopharyngeal swab relative to sample collection (days diagnosed pre-collection), COVID-19 symptoms expressed and overall disease severity

Patient ID #	Age (years)	Diagnosis relative to sample collection (days)	COVID-19 symptoms experienced by patient							Disease severity
			Fever	Body aches	Loss of smell	Loss of taste	Fatigue	Respiratory (cough, shortness of breath)	Headache	
1	24	6								Asymptomatic
2	24	6	X	X						Mild
3	48	28	X	X				X		Moderate
4	28	14	X		X	X				Moderate
5	24	21			X	X	X		X	Moderate
6	23	21	X				X		X	Moderate
7	24	28		X	X	X		X		Moderate
8	24	28	X	X		X		X	X	Moderate
9	23	16	X				X	X		Moderate
10	34	2	X	X				X		Moderate
11	53	2	X		X		X			Moderate
12	57	1	X		X				X	Moderate
13	39	4	X	X						Moderate
14	44	2	X	X	X					Moderate
15	56	2	X	X	X	X	X			Moderate
16	54	3	X		X	X	X			Moderate
17	54	3	X						X	Mild
18	32	4	X	X	X					Moderate
19	29	3	X	X	X			X	X	Moderate

Results from qRT-PCR assays for all samples were negative for SARS-CoV-2. Using serial dilutions of the positive control Sars-CoV-2 USA WA1 N-gene transcript RNA, the assay used can reliably detect 20 copies of Sars-CoV-2. Results from spiking positive control assays with RNA from extracted samples showed no substantial inhibition.

## Discussion

SARS-CoV-2 first was diagnosed in December of 2019 in Wuhan, China, and was detected in the USA in February 2020, rapidly growing to its existing pandemic status.

Extensive research continues to elucidate the locations of the SARS-CoV-2 in the human body and its modes of transmission. The primary route of infectivity is widely accepted to be respiratory, as the virus has been isolated from upper respiratory tract specimens and bronchoalveolar lavage fluid, and this is the primary means of detection. However, virus RNA has been detected in blood, urine, and stool specimens. It is currently unknown if active virus exists in extrapulmonary sites.

Our pilot study examined semen for SARS-CoV-2 RNA, including men with a recent diagnosis of COVID-19 as well as those in the convalescent phase. Irrespective of symptoms at the time of semen collection, no sample tested positive for the virus.

A study by Li et al. examined specimens from twenty-three patients and found that six had SARS-CoV-2 RNA in their semen [6]. Of the six patients, four were acute, and two were recovering from the virus. This finding was well covered by the media and was said to be proof that SARS-CoV-2 could be transmitted sexually. Though further clarified that rt-PCR was used for testing, the methodology for this study was not published in detail, therefore speculation about specimen collection and bias exist. For example, it is possible that contamination of semen samples with saliva or respiratory secretions might results in positive results for Sars-CoV-2.

Analogous to our study, Kayaaslan et al. found no evidence of viral RNA 16 in the semen of patients with acute SARS-CoV-2 infections and Song et al. found the same results in a study of 12 men in both the acute and recovery phase [7].

The presence of SARS-CoV-2 in semen has been difficult to study, particularly in patients with severe infections due to the severity of illness. Most patients with severe infections are not healthy enough to provide a semen specimen and are not typically seen in the medium of assisted reproduction clinics, though emergent or even posthumous sperm procurement for the purpose of eventual-assisted reproduction would validate future studies to be performed on this population. One weakness of our study is that no severely infected men participated; therefore, we cannot exclude the potential that semen from these patients could harbor the virus.

Based on the data from our study and others, we believe it is highly unlikely that SARS-CoV-2 exists in semen from infected men that have not been hospitalized or in those who have recovered, irrespective of symptom severity in these populations.

We refrain from making determinations on the ability for SARS-CoV-2 to be sexually transmitted, as though we find compelling reason to determine in mild to moderate disease there does not seem to be good evidence to support the presence of virus in semen, the leap to assuming that semen is the only form of transmission through sexual contact would be invalid. As noted earlier, there are many potential modes of transmission, and though semen would seem less likely to confer infectivity, intercourse would expose multiple different forms of transmission and as such conclusions regarding sexual transmission would be unsubstantiated at best, irresponsible at worst.

Given our small sample size, further studies are needed to determine conclusively if SARS-CoV-2 can be present in semen, particularly in men with severe symptoms from COVID-19.
